# The Foreign Journals: Medical Jurisprudence and Toxicology

**Published:** 1839-10

**Authors:** 


### MEDICAL JURISPRUDENCE AND TOXICOLOGY.

*On the Action of Arsenious Acid, and the Dose in which it may prove fatal to Man.* By Dr. Lachèse, Jun.

[The following is an abstract of the cases upon which the opinions of the author are founded.]

Case i. On the 5th August, 1830, P—— went to the house of his uncle T——, and remained some time, sitting close to the vessel which held the domestic store of salt. The following day, T., his wife, and three other persons had some soup for dinner. Very shortly after the meal, they all suffered more or less from oppression at the stomach, an acrid sensation in the throat, general uneasiness, nausea, and vomiting. At supper the remainder of the soup was consumed, the symptoms reappeared with increased violence, and lasted throughout the night.

On the 7th, the whole of the party felt a burning heat in the stomach and throat, but there was no vomiting. The wife went to her work; and as she did not take her meals at home, she remained that day free from any illness. On the 8th, three of the party left home, but complained of lassitude and weakness of the limbs. After their dinner, on their return, they were again seized with vomiting, and T., with another of the family, suffered severely. They complained of violent pain in the stomach, and were attacked with frequent vomiting of biliary matter mixed with food. T. suffered from severe pain in the legs, so that he could not stand,—vertigo, and convulsions. On the 26th September, he died; his lower extremities having become completely paralyzed before death. The other person, a female, died on the 18th October. No inspection of the bodies was made. It is worthy of remark, that all who came to the house during the illness of these persons, and who partook of any food, suffered from similar symptoms. After a number of trials, the salt was thrown away, and the sickness disappeared.

CASE II. The wife of the deceased T., who had not suffered so much as her husband, was gradually recovering, when, about the end of August, P. made her a present of some plums, desiring her to eat them. She ate a few, and in ten minutes afterwards was seized with violent colic, vomiting, convulsions, and insensibility. For several days her life was despaired of; but she left her bed at the end of eight months, much enfeebled in body. She was worn and emaciated, suffered from constant gastrodynia and indigestion. Two other persons, who merely tasted these plums, were almost immediately seized with vomiting, which relieved them.

CASE III. On the 24th July, 1832, Pl——, who had inherited one half of the property of his uncle T——, paid a visit to his brother-in-law, M——, the inheritor of the other half; and after having several times examined some flour in a bin, left the house. On the 26th, the wife of M. baked, as usual, a hundred pounds of bread, and gave some of it to two of her neighbours. On the same day, about noon, thirteen persons partook of this bread, and soon afterwards they all suffered from symptoms of oppression and pain in the stomach, nausea, vomiting, accompanied by an acrid, burning sensation in the throat, colic, pain in the limbs, thirst, and general uneasiness. On the following morning, some of these persons, not suspecting the cause of their illness, breakfasted with the same bread. The symptoms reappeared in an aggravated form, in some with diarrhœa, in others with obstinate constipation. They also suffered from vertigo and general weakness. They recovered, after having been obliged to keep their beds for a few days.

CASE IV. On the 24th June, 1835, V—— took for supper some food prepared by his wife. He died in *five hours* afterwards, having suffered from vomiting, intense thirst, and excessive weakness. On inspection, arsenic, in very fine powder, was discovered in the alimentary canal, the upper portion of which was in a state of intense inflammation. Near the ileo-cæcal valve some white semifluid masses were found, which were thrown away without examination.

CASE V. On the 23d June, 1832, a young woman ate some bread, on which a quantity of coarsely powdered arsenic, mixed with butter and rice, had been secretly spread. In *two hours* she was seized with vomiting, diarrhœa, and severe pain in the stomach. On the 25th she was seen by a physician. Her features were altered, her eyes sunk, and her pulse small and irregular; the abdomen was tense and painful, the limbs cold, the arms and hands in continual motion. She died in the evening of that day, more than fifty hours after having taken the poison.

Dr. Lachèse then observes that an analysis of the cases of these twenty persons, who were certainly poisoned by arsenic, will enable us to resolve these two important questions: 1. *What is the action of arsenic?* 2. *In what dose does it begin to be poisonous?*

First. *What is the action of arsenic?* After adverting to the opinions of Brodie respecting the absorption and circulation of this poison in the blood, with its remote influence on the nervous system, the author attempts to settle, from the preceding facts, in what cases the alimentary canal, and in what the nervous system is liable to become more especially affected.

When arsenic is taken but once, and in a small dose, the stomach is but little affected. There is, perhaps, a sense of oppression in the stomach, an acrid sensation in the œsophagus, vomiting ensues, and the symptoms vanish. This was observed in the two individuals who tasted the plums in Case II. In a stronger dose, there will be nausea, vomiting, and colic, weakness of the limbs, and a feeling of lassitude, which may last several days. These were the symptoms manifested by the persons who had partaken of the bread, (Case III). The alimentary canal was here almost exclusively affected, but still there were traces of an incipient affection of the nervous system. If a still more powerful dose be administered *once* or *twice*, there will be, in addition to the gastro-intestinal irritation just described, pain in the limbs, general uneasiness and restlessness, with convulsive spasms of the muscles. The action on the nervous system is here more strikingly indicated; and to this must we ascribe the principal phenomena. For an illustration of this state, the cases of M. and his wife (Case III.) may be referred to, they having twice partaken of the bread which had been baked the day before. If a very powerful dose of arsenic has been given, the affection of the nervous system is as speedily induced as that of the intestinal canal; but the nervous affection is far more serious, and is evidently the cause of death, or, if the individual survive, of the chief symptoms under which he labours. The case of the woman T. (Case II.) affords an illustration of this kind of action.

Sometimes the operation of arsenic, in a large dose, is slowly manifested, a circumstance which would appear to be due to the state of the poison. In all the cases in which it was exhibited in fine powder, the progress of the symptoms was extremely rapid; for in all the individuals poisoned by P., and in the case of V. (Case IV.), who only survived five hours, the symptoms manifested themselves in a few minutes after the poison was swallowed. On the other hand, in Case V., the deceased swallowed the poison in the form of a coarse powder; she was not taken ill for two hours, and she survived its effects fifty hours. In a case related by M. Laborde, a girl continued to bite, during the greater part of the day, a lump of arsenic, which had been given to her. For several hours no symptoms appeared; and she did not die until after upwards of ten hours' suffering. In these two cases the arsenic was in lumps. Lastly, when small doses of the poison are successively administered for several days, the stomach and intestinal canal appear to be exclusively affected; and death may ensue from extensive morbid changes in these organs. The affection of the nervous system, although it may be observed especially at the beginning of the symptoms, is very slight. This view is borne out by a reference to the cases of the two persons who died in Case I. The following are the conclusions of Dr. Lachèse:

1. When arsenic is administered in a dose sufficient to prove fatal, it acts by suspending the functions of the heart and brain.

2. This action is more speedily manifested, in proportion as the arsenic is finely powdered.

3. When the poison is given in small dose, its action is confined to the stomach; and as the quantity is increased, so does the whole of the alimentary canal become affected. At the same time, the affection of the nervous system manifests itself with greater rapidity and intensity, the symptoms becoming more strongly marked, as the action of the poison is more powerful.

4. Several small doses of arsenic, given successively, produce slow poisoning. Death, in such cases, appears to be a result rather of extensive morbid changes in the alimentary canal than of any derangement of the nervous system.

Second Question. *In what dose does arsenic begin to be poisonous?*

In the facts above reported, there would have been nothing to guide us to the solution of this question, had it not happened, that the bread, mentioned in Case III., was submitted to careful analysis by experienced chemists. Several of those who analyzed it, thought that the proportion of arsenic was about half a grain to each pound of bread. This would give a ratio of one quarter of a grain to half a pound, and one eighth of a grain to a quarter of a pound. From the enquiries made, it appears that the smallest quantity of bread eaten by any of the party was about a quarter of a pound, from which it may be inferred that these persons had taken one eighth of a grain of arsenic. From this somewhat doubtful estimate, we may now proceed to judge of the symptoms respectively produced, in relation to the quantity of the poison.

One eighth of a grain of arsenic, mixed with three or four ounces of bread, by a reference to these cases, would produce no other effect, in a healthy adult, but that of speedy vomiting. This quantity of the poison does not appear to affect the mucous membrane of the stomach, or to become absorbed. It seems to give rise to a sudden anti-peristaltic motion in the stomach, by which its contents are expelled. When, at a meal, from half a pound to a pound of bread was eaten, and therefore from a quarter to half a grain of arsenic had been taken, the symptoms were more decided, and began to partake of the character of true poisoning. When this dose was repeated on the following day, the symptoms acquired still greater intensity, and with the usual severe disturbance of the stomach and bowels, there was considerable disorder of the nervous system. Four successive doses, i. e. from one to two grains of the poison, would give rise to gastro-enteritis, and such an affection of the nervous system as would be sufficient to destroy life. These conclusions are further corroborated by the following case.

CASE VI. On the 20th May, 1836, a young man, for the purpose of curing himself of a cutaneous disorder, took one eighth of a grain of arsenic instead of one sixteenth. This was the first time he had taken arsenic. In about a quarter of an hour after he had swallowed the dose, he experienced a sweetish but somewhat acrid sensation in the mouth, extending to the fauces. Ten minutes afterwards he passed a motion, and twice attempted to vomit, but without effect. In two hours he had lost all these symptoms.

Larger doses may, it is true, be given to individuals under medical treatment, without evil consequences; but this is usually accomplished by beginning with very small doses, and gradually increasing them. One half grain of arsenic, exhibited in a day, has been known to produce an illness of eight days; and it is stated by Monro, that a man was killed by a *quarter of a grain*, which had been prescribed for him by a quack. As an answer to this question, we may say that in a healthy adult one eighth of a grain of arsenic may give rise to a disturbance of the system; that from one quarter to half a grain would produce symptoms sufficiently severe to constitute true poisoning; and that in a dose of from one to two grains it may suffice to destroy life.

[REMARKS. We cannot allow this paper to pass without offering a few comments upon it, as the subjects of which it treats are of considerable importance in a medico-legal view. The cases, although short and somewhat imperfectly given, are nevertheless interesting. Case IV. is remarkable for two circumstances; the short period within which death took place (*five hours*), and the fact of the mucous membrane of the alimentary canal having been found *intensely inflamed*, notwithstanding the rapidly fatal effects of the poison. One striking point in these cases is that in all, with the exception of the young woman, Case V., the symptoms appeared very speedily, i. e. *a few minutes* after the poison had been swallowed. In the case which constitutes the exception, they did not show themselves for two hours, which is beyond the average time for their appearance Dr. Lachèse is, we think, somewhat hasty in drawing the inference that the action of the poison is more speedy in proportion as it is finely powdered. The symptoms, we are ready to admit, are more generally retarded where the poison has been taken in lumps or in a coarse powder, than where it has been taken in a finely divided state; but the fatal effects of the poison are sometimes more speedily manifested in the former than in the latter case. We have known death take place within a shorter period, where the poison was swallowed in lumps, than where eight times the quantity had been taken in powder. We do not attempt to draw any inference from a solitary case; and we think after all, the difference in the state of the poison will be found to modify the time of access of the symptoms as well as their nature, and not the *period* at which it may prove fatal to life. Dr. Lachèse concludes, from these cases, that the affections of the alimentary canal and nervous system appear simultaneously, and are in a direct ratio to each other, when a strong dose of the poison has been taken. We cannot join him in this conclusion. The case to which he refers (Case II.), in illustration of his opinion, merely proves the slow after-effects of arsenic when it does not prove fatal. It is rare that these two classes of symptoms commence and go on together. In general, it has been observed that the symptoms of nervous irritation do not make their appearance until the derangement of the alimentary canal has subsided; and although the two affections may become occasionally mixed and combined, yet we see no instance of this, in the cases reported, sufficiently well marked to justify the inference of the reporter.

Dr. L. does not appear to us to have succeeded in solving the second question, as to the dose in which the poisonous action of arsenic commences. By analysis, it appears that half a grain of the poison was found in a pound of bread; and although the Doctor allows this to be a doubtful estimate ("*une estimation peu certaine*,") yet he proceeds to reason on it, as if these facts were well established: 1, that each loaf contained an equal quantity; 2, that each quarter of a loaf contained an equal fractional portion; and 3, that the quantity of bread eaten, by each individual, was accurately determined. Having remarked the nature of the symptoms in these cases, he connects them with the quantity of bread, and therefore with the quantity of poison taken; and applying this reasoning to his other observations, he draws the inference of the quantity of poison taken in those unknown cases from the nature of the symptoms. We need scarcely remark, that this method of reasoning is not exactly adapted to aid in the solution of a medico-legal difficulty. Allowing that the poison existed in the proportion ascertained by the analysis of a pound of the bread, not more than fifty grains could have been thrown into the flour. But is it at all probable that these fifty grains should have been so kneaded into the dough, in the ordinary process of making bread, as that each pound should have received its half grain, and each quarter of a pound its eighth of a grain? We confess we cannot view this as in the least degree probable; and therefore we may ask what becomes of the author's conclusions respecting the specific effects of specific doses of the poison? Besides, the report shows that the analysts did not agree in their results; and, therefore, there does not appear to us to have been any just ground for the author's estimate in respect to the quantity of poison taken. Case VI. does not establish more than was known before; and with regard to the case quoted from Monro, as to the fatal effects of a *quarter of a grain* of arsenic, we have some doubts of its authenticity. The author should have given a reference to it. Although we fully agree with Dr. Lachèse as to the general effects of small doses of arsenic, and the quantity required to prove fatal in a human adult, yet it is not in consequence of the case in which he supposes the proportion of poison to have been analytically ascertained.] *Annales d'Hygiène. Avril,* 1837.

---

### *On some New Signs of Suspension having taken place during Life.* By M. Devergie.

In a memoir presented to the Academy of Medicine, M. Devergie notices two circumstances which, in cases of hanging, will prove whether suspension has taken place during life or not. The facts of an ejaculation of sperm in the last moments of life, in cases of hanging, and of the existence of spermatic animalcules in urine, when an emission of urine has immediately followed an ejaculation, are well known, and have led M. Devergie to search for these animalcules in the urethra of persons who have been found hanging. If in such cases the urethra be slit open, or, better still, if its contents be pressed out into a watch-glass, we find a mucous matter, more or less thick, exhaling a strong odour of semen, and containing here and there the peculiar animalcules which are found in the human spermatic fluid alone. But the place of these is occasionally supplied by a number of small rounded bodies resembling the animalcules without a tail; these M. Devergie conjectures, may be spermatic animalcules in an imperfect or rudimentary state. However that may be, the presence of semen in the canal of the urethra is a certain sign that suspension took place during life. The second circumstance is that the end of the penis is so reddened and moistened by a mixture of semen and mucus as to give the idea of a gonorrhœa having existed; whilst the *corpus cavernosum* and *spongiosum* are so filled with thick black blood as to form a striking contrast to the paleness of the same parts in cases of natural death. This sign is of as much value as the existence of sperm in the urethra, and is observed with greater facility. *Bulletin de l'Académie. Nov.* 20, 1838.

---

### *New Process for the Separation of Arsenic in Organic Mixtures.* By L. Malle, M.D.

Numerous experiments having convinced M. Malle that the ordinary processes (those of Hahneman, Rose, Fischer, Orfila, Rapp, and Taufflieb,) resorted to for the detection of arsenious acid in animal matters are more or less ineffectual when the quantity of poison does not exceed one tenth of a grain, he was led to investigate the subject closely, and now proposes the following method, as superior to those hitherto employed. The suspected matters, whatever be their nature, are to be placed in a porcelain capsule; any vegetable poison they may contain having been previously dissolved with alcohol and ether: a solution of hydrosulphate of ammonia is then poured on them, in order to transform into sulphurets the arsenic acid and any other metallic preparations capable of undergoing such transformation which may chance to be contained in the mixture. The liquid is then slowly evaporated, and the residue treated with alcohol saturated with ammoniacal gas, in order to precipitate organic substances, and dissolve the sulphuret of arsenic. The contents of the capsule are next filtered, whereby a fluid, containing alcohol, ammonia, and the sulphuret in a state of solution, is obtained. The whole is next placed in a retort, communicating with a mattress, and then heated in a water-bath, so as to distil over the alcohol and volatilize the ammonia. The residue is then treated with nitric and a little hydrochloric acid, in order to destroy organic substances, and convert the sulphuret into arsenic acid and sulphuric acid. Once this is effected, nothing remains to be done but to separate the two acids by adding some ammonia and ammoniacal sulphate of magnesia to the liquid, which precipitates the arsenic acid in the form of an ammoniacal arsenite of magnesia. This precipitate is collected on filtering paper, and, after having been carefully washed, is reduced by exposure to a current of hydrogen gas, in a small tube, drawn to a fine point, as recommended first by Berzelius.

By this process M. Malle was enabled to obtain a metallic ring from half a pound of alimentary substances, to which five milligrammes* of arsenious acid had been added; in order to remove all doubts of the nature of the metallic substance obtained, it was placed in a small vessel, full of dry oxygen, and thus converted into arsenious acid, which again was precipitated with hydrosulphuric acid in the form of a sulphuret, soluble in ammonia. *L'Expérience.* No. lxxxi. *Janvier,* 1839.

---

### *On Hydrated Peroxyde of Iron (Ferri Subcarbonas) as an Antidote to Arsenic.* By a Commission of the Academy of Medicine of Paris.

[In our First, Fourth, and Seventh Volumes, some accounts were published of the facts which seemed to prove the value of peroxyde of iron as an antidote to arsenious acid. The case in the Seventh Volume was one in which a patient that had taken arsenic was rescued from apparently certain death by the administration of this compound by Dr. Deville; and the subject was then deemed of such importance by the Academy of Medicine of Paris, that a commission was appointed, composed of MM. Deville, Sandras, Nonat, and Guibourt, to enquire into the value of the supposed antidote, and the best means of preparing and administering it. The following is an abstract of the therapeutical part of the Report which has been presented.]

* The milligramme is = ·0154 English grains.

Dogs were first destroyed by arsenic, to determine the time in which that poison proves fatal when its action is not interfered with, and to obtain a standard by which the beneficial effects of the antidote might be more nearly estimated. Other dogs were killed by ligature of the œsophagus, to determine the influence which that necessary operation would have in their destruction. Four different oxides of iron were then used with different dogs, to whom arsenic had been given. A moist protoxyde, containing 19 per cent. of anhydrous peroxyde, and a black oxyde containing 7 per cent. were entirely inefficacious; the dogs died as soon as they would if no means had been used to save them. The moist hydrated peroxyde, and the common dry hydrated peroxyde of iron (the subcarbonate of iron of former pharmacopœiæ, and ferri-sesqui-oxydum of the present,) both produced more important results. Ten animals poisoned with arsenic were treated with the first. In the first experiments large doses (half a drachm) of arsenic were given, and comparatively small quantities (four, six, or eight ounces) of the magma of peroxyde of iron; yet all the animals lived many hours longer than when the action of the arsenic was left uncontrolled. In the subsequent experiments, twelve grains of arsenic were given, and from eight to sixteen ounces of the antidote; and all the dogs thus treated lived as long as the ligature of the œsophagus would permit them.

With the second compound (the subcarbonate or sesqui-oxyde of iron), in three experiments, in which four grains of arsenious acid and three ounces of the antidote were given, the animals, like those last mentioned, lived to the full period which is possible after tying the œsophagus. The arsenite of peroxyde of iron (the salt which was supposed to be found in the stomach in the preceding cases) was next given to dogs, and evidently acted as a virulent poison. But the apparent anomaly of these cases was proved, by some further experiments, to result from the arsenite of iron, thus administered, being decomposed by the hydrochloric and lactic acids of the gastric secretion, and some uncombined arsenious acid being set free; and this showed that, to counteract the effects of the arsenic, it would be necessary to administer the peroxyde of iron in considerable excess, so that every portion of arsenious acid, whether originally existing in the stomach or set free by the decomposition of arsenite of iron, might be completely neutralized.

The results of all these experiments on animals were completely confirmed by the chemical examinations by which M. Guibourt determined the composition and the mutual actions of the substances employed. Thus the first two oxydes that were employed were of no avail against the effects of the arsenic; and from M. Guibourt's chemical researches it was evident that they did not render arsenious acid insoluble. In the physiological experiments the moist hydrated peroxyde of iron neutralized the arsenious acid in the stomach, imperfectly, indeed, when there was a proportionally large quantity of acid, but completely when the proportion of arsenic was small and that of the peroxyde considerable; and in the chemical experiments it appeared that ten parts of the dry peroxyde were necessary to neutralize one of arsenious acid. In the physiological experiments, again, the dry peroxyde completely neutralized the arsenious acid; and in the chemical the same peroxyde reduced to traces scarcely perceptible the precipitate of arsenious acid that could be obtained by Marsh's apparatus.

It is evident, then, (the Report continues,) that in the circumstances which have been determined, dogs have taken arsenic sufficient to destroy them in a few hours, and that yet they have lived as long as if only the œsophagus had been tied. The poison, therefore, has had no effect;—the peroxyde of iron, which we have employed, is, consequently, a real and efficacious antidote to arsenious acid.

Now, it is demonstrated that we have *disempoisoned* dogs that had taken arsenic; we have determined what doses of hydrated peroxyde of iron are necessary to neutralize fatal quantities of arsenious acid; we have employed, with the greatest success, not only the moist peroxyde of iron (always an inconvenient compound), but also the dry hydrated peroxyde, so common in the shops under the name of subcarbonate of iron, and so easy to administer in all imaginable doses, and so little injurious that we can see no necessity to limit the employment that may be made of it in these cases of poisoning. The following, then, is the mode of treatment which we should advise to be employed in these cases.

The first duty of the physician is to remove from the stomach the greatest possible quantity of the poison by vomiting. For this purpose watery drinks are improper; but while waiting for the peroxyde of iron, the doctor should tickle the vula, and administer oil, which will not dissolve the arsenious acid; but above all, as soon as it can be procured, the patient should be gorged with warm water, charged with some ounces of the peroxyde. The water will cause vomiting, and the peroxyde of iron, which is suspended in it, will neutralize the particles of arsenious acid that are dissolved. This means fulfils at once the two chief indications in every case of poisoning. The antidote must be given as soon as possible, and in such a quantity and for such a time that there can be no reason to suppose a single atom of arsenious acid remains in the stomach. Four ounces of the dry hydrated peroxyde of iron (the subcarbonate of iron of the shops, the ferri-sesqui-oxydum of the London Pharmacopœia,) should be suspended in twenty-four ounces of water, and a good glass of the mixture should be taken every ten minutes. After four ounces are consumed, fresh doses of the same compound should be administered in the same way, and the patient should not be considered out of danger till he has taken at least half an ounce of the peroxyde for each grain of arsenious acid supposed to have remained in the stomach. If, afterwards, symptoms of inflammation should manifest themselves, prompt recourse must be had to antiphlogistics and other appropriate means.

In the short discussion which followed the reading of the Report, M. Devergie said he thought the conclusion too explicit; that the large quantities of the peroxyde which it was necessary to administer, even when but a small quantity of arsenic had been taken, would always be an obstacle to its use, and that it would be unwise to let the presumed antidote take the place of any of the other means usually resorted to, especially continued vomiting. At the conclusion of the discussion it was agreed to employ the words "           antidote," instead of "real and efficacious antidote;" and the Report was then unanimously ordered to be printed. *Revue Médicale. Mai et Juin*, 1839.

